# Supplementary material for: Humans underestimate the movement range of their own hands
Source: Commun Psychol. 2024 Nov 1;2:104. doi: 10.1038/s44271-024-00153-x (PMC11530695; doi:10.1038/s44271-024-00153-x)
Supplement: Supplementary file 2 — Supplementary Materials [file 44271_2024_153_MOESM2_ESM.pdf]

Supplementary Information

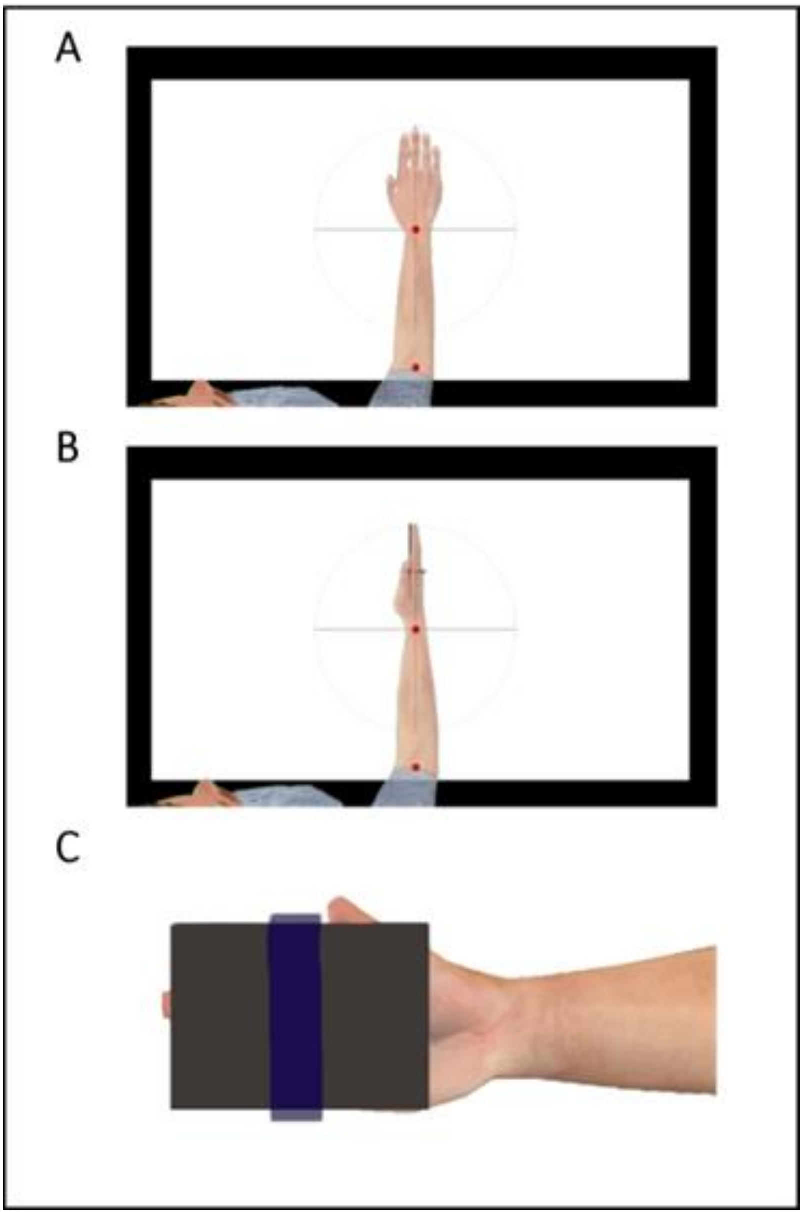

Supplementary Figure S1. Setup in Experiment 1.

Resting position of adduction and abduction (A) and of flexion and extension (B) movements. The red dots correspond to the position of the double-sided adhesive pads used to ensure correct positioning of participants' hand. Panel C depicts the hand-sized rigid plate attached to participants' hand with velcro (blue-colored) used in the flexion and extension conditions.

Supplementary Table S1

Means and standard deviations of real and estimated movement amplitudes in degrees across the type of movements tested in Experiment 1

Measure	Mean	SD	Hand	Movement type
Adduction	34.9	20.92	Dominant	Estimated
Abduction	34.7	18.06		
Flexion	66.0	37.36		
Extension	55.7	31.99		
Adduction	33.4	18.80	Nondominant	
Abduction	32.7	16.93		
Flexion	65.7	37.86		
Extension	55.0	32.34		
Adduction	30.0	7.97	Dominant	Real
Abduction	44.6	9.79		
Flexion	90.1	15.67		
Extension	67.2	10.32		
Adduction	30.9	7.90	Nondominant	
Abduction	44.8	9.80		
Flexion	88.2	18.46		
Extension	71.6	10.65		

Note. n = 59; units: degrees.
